# Highly dispersive multiplexed micromechanical device array for spatially resolved sensing and actuation

**DOI:** 10.1038/s41378-024-00816-z

**Published:** 2024-11-26

**Authors:** Leonardo Gregorat, Marco Cautero, Leonardo Vicarelli, Dario Giuressi, Alvise Bagolini, Alessandro Tredicucci, Giuseppe Cautero, Alessandro Pitanti

**Affiliations:** 1 Department of Engineering and Architecture, Università degli Studi di Trieste, Trieste, Italy; 2 Department of Physics, Università degli Studi di Trieste, Trieste, Italy; 3 Department of Physics, Università di Pisa, Pisa, Italy; 4 Elettra Sincrotrone Trieste, Trieste, Italy; 5 Center for Sensors and Devices, Fondazione Bruno Kessler, Trento, Italy; 6 NEST Lab, CNR - Istituto di Nanoscienze and Scuola Normale Superiore, Pisa, Italy

**Keywords:** Electrical and electronic engineering, Electronic properties and materials, Electronic devices

## Abstract

The powerful resource of parallelizing simple devices for realizing and enhancing complex operations comes with the drawback of multiple connections for addressing and controlling the individual elements. Here we report on a technological platform where several mechanical resonators can be individually probed and electrically actuated by using dispersive multiplexing within a single electrical channel. We demonstrate room temperature control of the individual device vibrational motion and spatially-resolved readouts. As the single elements have proven to be excellent bolometers and individual nodes for reservoir computing, our platform can be directly employed for single-channel addressing of multiple devices, with immediate applications for far-infrared cameras, spatial light modulators and recurrent neural networks operating at room temperature.

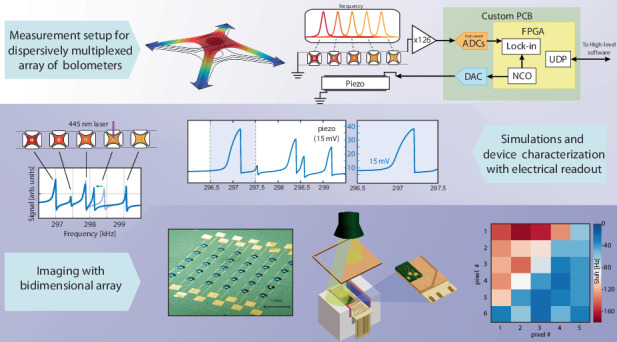

## Introduction

One of the most tracked routes for obtaining devices capable of complex operations is arranging relatively simple elements in organized networks. Among the impressive technological examples of multi-devices networks one can find methods for scaling-up the computational power of electronic devices, as currently done in most computer architectures (see, for example ref. ^1^), combining information processing in complex, multiparametric nonlinear systems^2^, or realizing probes which spatially encode the detected signal, as done for example in charged-coupled devices (CCD) cameras^3^. However, addressing single elements in a complex network requires non-trivial technological efforts, with possibilities including coupling each device to different input/output channels (see for example the 96×96 torsional MEMS matrix array in ref. ^4^) or arranging them in ordered matrices, which can be read employing shift registers^5^. In this context, the possibility of addressing different devices in arbitrarily arranged networks is highly desirable and will lead to both a strong simplification of current architectures and new paradigms. Dispersive multiplexed readout schemes—in which each device is characterized by a specific frequency—have already found application in cryogenic sensing arrays, including bolometers and calorimeters^6,7^, airflow sensing^8^, superconducting detectors^9,10^; more recently, a similar concept has been applied for reading-out qubits based on quantum-dots^11^ or coupled superconducting RF resonators^12,13^. Moreover, a fast sequential addressing of 20 individual frequency-encoded resonators for mass spectroscopy in cryogenic environment has been reported^14^. Although the readout and the devices presented there exhibit video-rate capabilities, their particular geometry would be ill-suited for particular applications, such as radiation imaging, due to the low fill factor of the active devices.

Some interesting solutions have been also reported in uncooled systems showing capacitive-based and near-field optical read-out of tens of elements arrays of nanobeams^15^ and of nanocantilevers^16^, respectively. Moreover, in terms of speed it is worth to highlight the demonstration of real-time multiplexed read-out of thermal motion of three optomechanical resonators addressed by a single optical waveguide^8^.

In this manuscript we illustrate a device array operating at room-temperature and composed by individually and electrically addressable micromechanical trampoline resonators (MTRs)^17^. Exploiting a smart design of their vibrational eigenfrequencies, we show how we can actuate and sense the state of individual devices using a single, dispersively multiplexed communication channel. In particular, we show a precise control of the motional state of individual MTRs. Local control can be employed for a new concept of spatially-encoded, 100 s kHz–10 s MHz modulators, capable of projecting non-trivially structured light beams, surpassing the current technology mostly based on liquid crystals^18^. We believe that our report shows (i) the first imaging reconstruction using a multiplexed read-out array at room-temperature and (ii) precise control of the coherent vibration of individual resonators, opening the avenue to an enhanced technology level whose main applications will be discussed below. Furthermore, while the open loop read-out of the whole presented 30 resonator arrays requires around 10 s, single pixels show a cut-off operation frequency of 10 s of Hz^19,20^, potentially rivalling the optomechanical-based real-time sensing^8^ in an all-electric platform. Furthermore, accessing the nonlinear motional regime through Duffing nonlinearities, MTRs can find usage as physical nodes for other recently emerging applications, e.g. reservoir computing system^21^, where low energy consumption and high operational frequency are key features^22,23^, or fast spatial light modulators^24,25^. Finally, we show the use of the matrix array as a single-channel imaging device, which can be operated in a wide spectral range (from the visible to the sub-THz range) given an appropriate design of the MTRs^20^.

## Results and discussion

### Matrix architecture

**Fig. 1:**
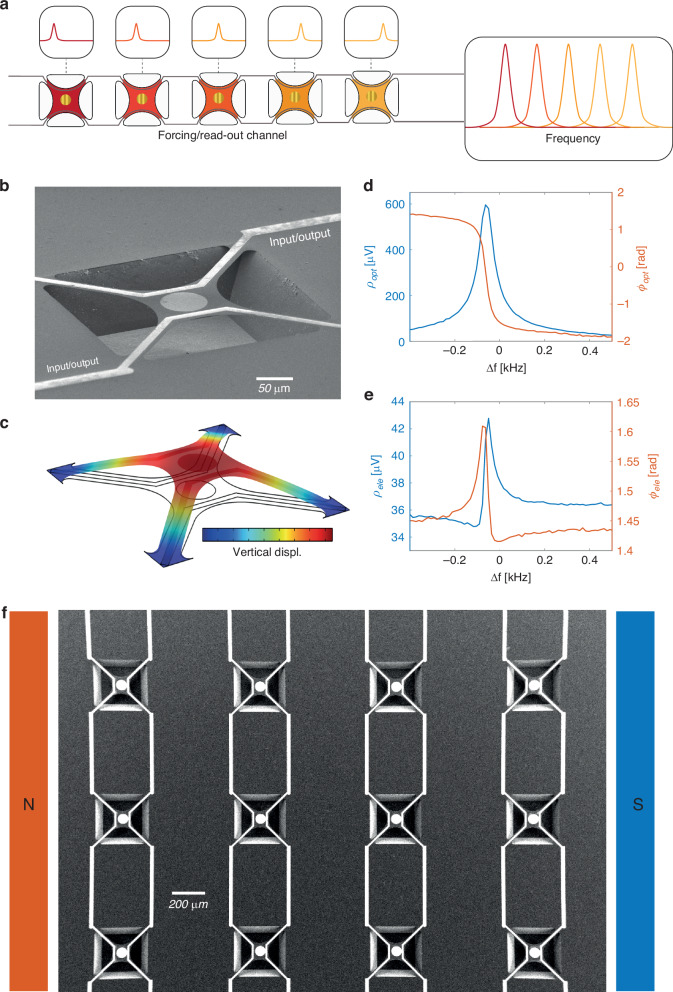
Working principle of the MTRs and dispersive multiplexing architecture. **a** Several devices with different resonance frequencies can be independently addressed using a single communication channel for readout or forcing. **b** SEM (tilted view) of a typical MTR, the mechanical resonance investigated is its fundamental vibrational mode simulated in (**c**). Spectra of the fundamental mode resonance measured by self-mixing interferometry (**d**) and magnetomotivevoltage (**e**). **f** SEM (top view) of a portion of a MTR array placed in a static magnetic field

The core idea in our approach is to exploit the different resonance frequencies of appropriately designed devices coupled to the same input/output channel (see Fig. 1a). Frequency-division multiplexing/demultiplexing on the channel allows one to actuate or read out single devices. Being the single elements arranged in a well-defined spatial configuration, we can obtain spatially encoded information or address pixels with a precise set of coordinates within the network. The single device from which we implemented the dispersive multiplexing scheme is a silicon nitride MTR^17^. These devices have shown interesting mechanical properties at room-temperature, including ultra-high mechanical Q-factors^26^ and fine control of the material effective parameters in a metasurface approach^27^, which can be extremely beneficial to sensing applications giving sharp mechanical lines to be used in transduction experiment, as demonstrated in preliminary results in a broad spectral range from visible to sub-THz^20^. Moreover, their large operating frequency and the possibility to engineer their interaction with electromagnetic radiation via surface functionalization, makes them strong candidates as modulators of all light parameters, from amplitude and phase to spin and orbital angular momentum^28,29^, surpassing current technologies based, for example, on slow liquid crystal arrays. A typical fabricated single-pixel device is depicted in the SEM image of Fig. 1b (details on the fabrication can be found in the Supplementary Section S1). Two metallic electrodes are placed on the membrane and work as input/output channels, as will be described later. The basic trampoline geometry we report here is tuned for realizing single pixels of a 5 × 6 camera operating in the visible spectral range: different parameters can be employed for different applications.

Regarding the mechanical motion, we considered the fundamental vibrational mode of the MTR, which showed a large out-of-plane displacement of the central membrane region as shown by the finite-element-method (FEM) simulation of Fig. 1c. The MTR frame/tether dimensions and the Si_3_N_4_ tensile stress were chosen in order to reach a mechanical frequency of around 300 kHz (details on the FEM simulations and MTR dimensions in Supplementary Section S3). Standard optical interferometry based on the self-mixing effect can be used to measure the device displacement, with a typical interferometric spectrum reported in Fig. 1d. Here the membrane was excited using a piezoelectric ceramic actuator placed underneath the whole chip. A lock-in amplifier was used to demodulate the self-mixing signal using the actuator drive as a reference and returning spectral information about the MTR’s amplitude and phase, showing a clear Lorentzian peak for the former and the typical associated π shift for the latter. More details on the optical interferometry setup can be found elsewhere^30^. Despite its clear signal, optical interferometry is not well suited for simultaneous characterization of multiple devices, whereas electrical signals are better candidates for parallelization and control. To this end, we employed a magnetomotive approach^31–34^ by exploiting the electrical contacts on the sides of the membranes and placing the whole chip in a static and planar magnetic field obtained with Nd permanent magnets (see Fig. 1f). The membrane’s vertical motion driven by the actuator changes the concatenated magnetic flux, giving rise to a magnetomotive voltage, as per Faraday’s law, which can be measured at the contact ends. Furthermore, the same contacts can be used alternatively as local actuators: forcing an AC signal on the wire, the MTRs can be actuated via Lorentz’s force, which is essentially directed out-of-plane in our setup geometry.

Employing a similar readout scheme as in the self-mixing case, we demodulated the magnetomotive voltage using the piezoelectric layer drive as a reference and obtained the results shown in Fig. 1e. Despite a change in the line shape which will be discussed later in more detail, the magneto-electrical readout clearly shows a high signal-to-noise ratio resonant peak corresponding to the membrane’s fundamental vibrational mode.

Multiple devices can be arranged in a network, as shown in Fig. 1f. In our architecture, each device has a different width of the suspending tethers, setting a different frequency for the mechanical mode and granting individual addressing. The matrix array we illustrate here is composed by 6 linear arrays each containing 5 single devices. Each linear array can be contacted separately or connected to the neighboring ones, in such a way that a long, single serpentine-shaped circuit is formed, running from the top-left corner device to the right-top one.

### Device characterization

**Fig. 2:**
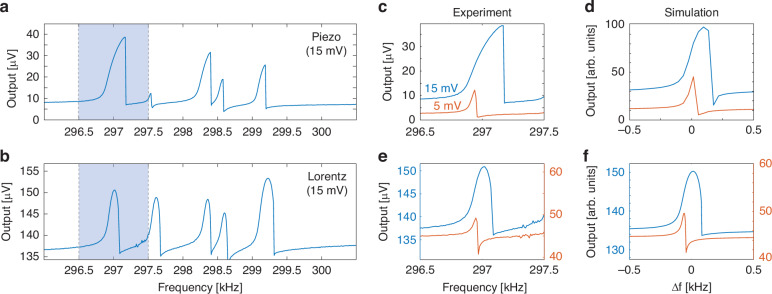
Characterization of the devices' electrical readout signal with different actuations. Signal of a 5-devices linear array using piezoelectric substrate (**a**) and Lorentz’s force (**b**) forcing. Comparison between experimental data (**c**–**e**) and simulations (**d**–**f**) for single peak resonances and with different driving voltages

Figure 2a, b show the electrical characterization of a single linear array, actuated using the piezoelectric layer and Lorentz’s force, respectively. The array is operated at room temperature and in moderate vacuum (pressure ∼ 10^ −2 ^mbar) to enhance the resonances’ quality factor. As can be seen, a ∼ 3 kHz span of the driving frequency highlights, on the single output channel, the presence of 5 different resonators. A simple model for the magnetomotive readout (Supplementary Section S3), taking in account our experimental setup, returns a displacement sensitivity of 9.3 nm/µV of detected signal, which is compatible with what was observed in previous reports with similar devices^27,30^. Considering this, it is possible to evaluate the maximum displacement of the membranes (e.g. ∼300 nm for the first peak in Fig. 2 (a)). The resonances’ displacement is far above the natural thermal fluctuation at room temperature, which can be estimated via the equipartition theorem^35^ as ∼ 10 pm_rms_, and therefore represents a negligible noise contribution.

Interestingly, the peak amplitude distribution is different for the two driving configurations, being each resonance slightly different in the piezo forcing, whereas more similar in the Lorentz one. This can be ascribed to local imperfections within the piezoelectric layer, the devices and in the mechanical coupling between the two, whereas the Lorentz-based forcing results are affected only by the device imperfections. Note that, if needed, the oscillation’s amplitude of an individual device can be tuned by piecewise custom driving waveforms (more on this later). The spectral shape of single resonators can be obtained by considering a toy-model dynamic equation system which, for example for the piezoelectric drive, reads as:

1 $$\left\{\begin{array}{ll} \ddot{x}_{P}(t)&=\,-\frac{{\gamma}_{P}}{2}{\dot{x}}_{P}(t)\,-\,{\omega}_{P}^{2}{x}_{P}(t)+\,{A}_{F}\sin ({\omega}_{F}t)\\\ddot{x}_{m}(t)&=\,-\frac{{\gamma}_{m}}{2}{\dot{x}}_{m}(t)\,-\,{\omega }_{m}^{2}{x}_{m}(t)-\,{d}_{m}{x}_{m}{(t)}^{3}+{{cx}}_{P}(t)\end{array}\right.$$

where x_i_, γ_i_, ω_i_ are respectively the generalized displacement, damping rate and resonant frequency for the piezo (i = P) and for the membrane (i = m). Additional terms include the piezo harmonic drive at frequency ω_F_ and amplitude A_F_, the piezo-to-membrane coupling coefficient c and the Duffing nonlinearity coefficient d. Note that here we have assumed unitary masses. The electrically detected signal is given by the sum of two contributions: the time derivative of the magnetic flux through the MTR and the crosstalk between the drive signal and the readout wire. The latter can be considered to have a constant phase and amplitude through the resonance peak due to the narrow resonances of the membranes (Q_m_ > 1000). Hence, the detected signal is expected to assume a Fano-like line shape due to the interference between the two contributions, further modified by the Duffing term at large driving inputs. Figure 2 (c) and (d) report experimental and simulated results for a selected device actuated by the piezoelectric layer at different driving voltages. The simplified model captures well the most relevant features of the resonances for both drives. Note that a significant asymmetric deformation caused by Duffing nonlinearity is already present at 15 mV drive. Similar considerations apply to the Lorentz forcing (details on the differential equations can be found in Supplementary Section S3): here the crosstalk between the forcing and reading electrodes is stronger, leading to a higher out-of-resonance signal and to a smoother shape in case of destructive interference due to the phase shift of the resonance. Even in this case we obtain a good qualitative agreement between experiment (panel (e)) and simulations (panel (f)) for both driving values.

### Device actuation

**Fig. 3:**
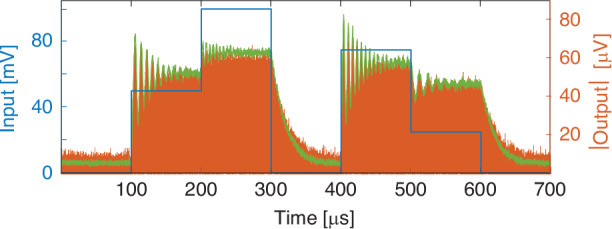
Actuation of a single MTR. Amplitude of the input at the MTR resonant frequency (blue), modulus of the MTR’s magnetomotive response (orange) and its envelope (green)

A strong feature of our system stems from the possibility of controlling the motional state of individual resonators by acting on a single input channel. An amplitude modulated single tone driving signal at the resonance frequency of a MTR is transduced by the motion of the resonating membrane. As an example, Fig. 3 reports a piecewise amplitude modulation function (blue) which is imposed on the sinusoidal excitation carrier of a MTR. The envelope (green) of the MTR’s output modulus (orange), once the electrical crosstalk has been removed, varies proportionally, albeit nonlinearly, with the excitation. A time series can then be encoded-in and decoded-from the membrane oscillations. As each MTR has a different resonance frequency, one can address one or more at the time using a linear combination of sinusoids as the driving signal. The switching speed, estimated around 30 ms and limited by the resonator’s Q-factor, makes the system appealing for video-rate operation, and can be further improved by changing the vacuum level.

**Fig. 4:**
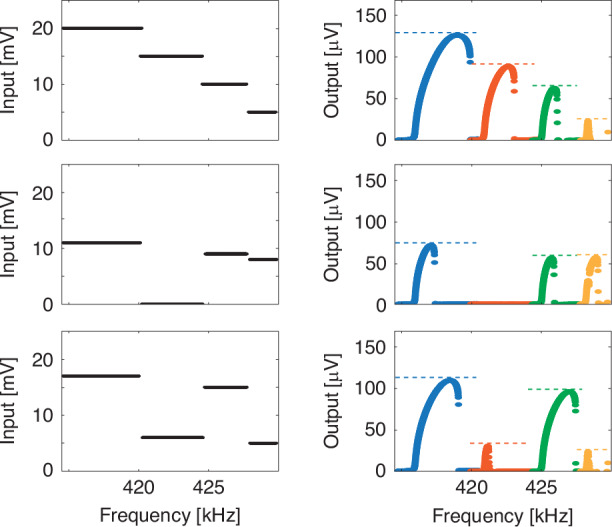
Independent actuation of multiple serially connected MTRs. Spectrum of three arbitrary amplitude modulated driving signals (left) and the corresponding spectral responses (right). The spectrum maxima have been dashed in the right panels for an improved input–output comparison

The strong nonlinear regime of operation at constant frequency in Fig. 3 is due to the Duffing contribution at high driving voltages (see Supplementary Section S4). Leveraging this nonlinearity, each MTR can be operated as a single node of a neural network and employed for hybrid^22^, or with (digital) delayed feedback^35^, reservoir computing. While with our current arrangement, where there is no mechanical coupling between the devices, only a digital delay feedback approach could be used, different MTRs arrangements can be imagined in such a way to create small clusters of coupled resonators. For example, by placing the devices on a common suspended frame; this would automatically grant an intrinsic feedback effect which would be directly embedded in the chip. Moreover, the individual control of different MTRs allows for parallel operation of several nodes, which can be simultaneously trained on different parameters of complex time series. To this end, Fig. 4 shows the excitation of 4 different MTRs within the same input channel using arbitrarily generated piecewise spectral functions. As can be seen, each resonator (second column) proportionally responds to the amplitude of the input signal (first column). In the weak nonlinear regime we are operating at, the input sequences are well transposed in the maximum of the output signal. whereas increasing the drive would produce more relevant nonlinearities.

### Linear and matrix imaging

**Fig. 5:**
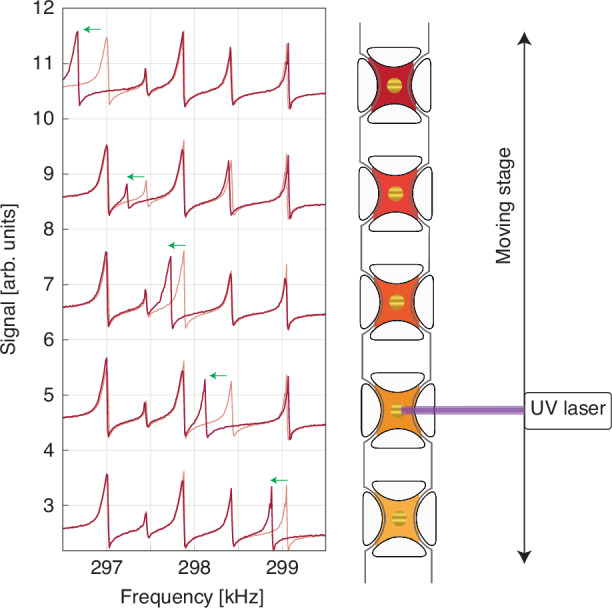
Single pixel sensitivity of a dispersively multiplexed linear array. Comparison of laser-off (light red) and laser-on spectra (dark red) when different MTRs on a linear array are illuminated by the UV laser

A direct use of the MTR matrix array is linked to distributed sensing and imaging. Recently, MTRs have revealed excellent properties as room-temperature thermomechanical bolometers, with broadband spectral operation at video-rate and noise equivalent power (NEP) as low as 7 pW/√Hz in the medium-infrared^36^ and 100 pW/√Hz in the sub-THz range^20^. The MTRs here investigated have a central metallic layer which is employed for light absorption in the visible to UV spectral range, being Si_3_N_4_ transparent in this frequency range. The detection capabilities of single MTR pixels have been tested by using a UV-laser pointer mounted on a translational stage. The laser had a wavelength of 445 nm and the spot on the sample surface was about 700 µm, with power of roughly 300 µW. When the laser was shined on a particular MTR pixel, it red-shifted its resonant frequency due to thermal expansion, without affecting the structure of the other resonances. This can be seen in Fig. 5, where the piezoelectrically-excited, laser-off spectrum (light red) can be compared with the laser-on one (dark red) when aimed at different pixels on the same linear array. The slightly different shifts of the pixels are due to the alignment conditions of the laser, which has been placed on the MTRs’ side to avoid resonance crossing in this particular chip. It is important to stress that, while the response of the devices follows a non-linear regime, the aforementioned frequency shift is linear with the power of the incident radiation as previously reported in ref. ^20^ and therefore mechanical nonlinearities do not affect imaging in open-loop configuration. Choosing a larger frequency spacing between the resonators could give a better dynamic range, increasing the maximum power at which neighboring device resonances overlap due to their shift, at the expense of a reduced number of devices and a wider mechanical spectral range to be interrogated.

**Fig. 6:**
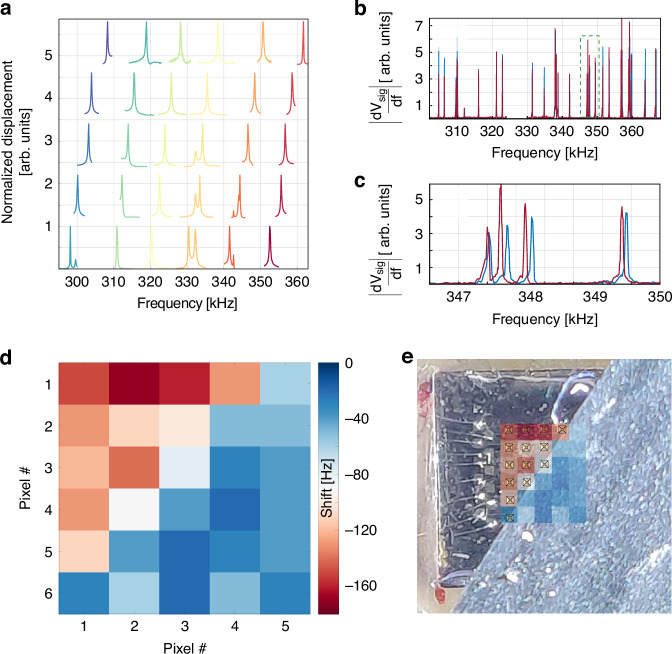
Characterization of an MTR array and visible light imaging principles and results. **a** Laser Doppler vibrometer characterization of the matrix array as fabricated. **b** Comparison of the piezo-excited dark (blue) and illuminated (red) spectra. The modulus of the signal derivative has been plotted for enhanced peak recognition. A zoomed plot on a selected frequency range is reported in (**c**). **d** Single channel imaging of a masked UV source. **e** Detected image superimposed on a photography of the masked device

Single channel readouts can be used to uniquely reconstruct the position of the illuminated pixel; the same concept can be expanded to the whole two-dimensional matrix for imaging applications. Figure 6 (a) reports a laser Doppler vibrometer characterization of our whole chip just after fabrication. The chip was composed by 30 pixels arranged in a 5×6 grid. As can be seen, each pixel has its own resonant frequency and can be uniquely identified by inspecting the mechanical spectrum. The imaging experiment has been performed by illuminating the chip with a RND Lab LED torch (700 lm placed at roughly 12 cm) and using an appropriate masking, covering in this case the lower right side of the matrix. The electrical characterizations of the complete, piezo driven, matrix in the dark and illuminated are reported in Fig. 6 (b); here we plotted the modulus of the signal derivative, |dV_sig_/df |, for enhanced peak recognition. The sharp edge produced by the non-linear vibration provides us with a high modulus of the derivative for easier resonance frequency estimation. As can be seen, especially in the zoomed-in frequency range reported in Fig. 6 (c), the resonant peaks’ position upon illumination (red line) are shifted by different amounts with respect to the ones in the dark spectrum (blue line). As can be inferred from the slightly different frequency range in panel (a) and (b), the resonance of single devices experienced a nonuniform frequency shift mostly attributed to ambient conditions and device aging (e.g. due to plastic deformations in the gold layer and Cr/Au interdiffusion). Upon remapping of the frequency-to-pixel position, we obtained the map in Fig. 6 (d), which clearly shows the masking, with a strong frequency shift found on the illuminated upper left side of the matrix. This can also be better appreciated in panel (e), where the resulting image has been superimposed on the masked device photography, where the single pixel position has also been indicated. The operational speed of our imaging device is mostly limited by thermal relaxation dynamics, which still allows for single device operation at video-rate^20^, thanks to the large thermal conductivity of the gold contact which compensates the high thermal resistance of silicon nitride. Adding the possibility to operate the MTRs as broadband bolometers, up to the sub-THz, makes our platform of interest for efficient imaging devices in the far-infrared spectral range operating at room-temperature.

## Conclusions

In this manuscript we illustrate a technological platform where a matrix array of MTRs can be operated, at room temperature, employing a single input/output electrical channel. Exploiting the dispersive multiplexing, which is a powerful tool in properly designed cryogenic electronics systems, we reconstructed a masked image in the visible spectrum. Room-temperature operation and the versatility of the MTRs as thermomechanical infrared bolometers^20,36^ make our platform of extreme interest also for THz and sub-THz imaging cameras for laboratory and in-field operation. The additional feature of manipulating the motional MTR state at a single pixel level opens the route to new paradigms for MEMS network arrays, like novel spatial light modulators or parallel platforms for reservoir computing, where low-cost training can be combined by parallel operation on several nodes composed by single or a cluster of micromechanical resonators.

## Methods

### Electrical readout

The electrical readout is performed with a custom FPGA-based lock-in amplifier, to obtain both time and frequency domain measures with arbitrary amplitude and frequency modulation capabilities. The frequency domain electrical readout was also performed with a frequency sweep capable lock-in amplifier (Zurich Instruments UHFLI) with comparable results.

### Time domain processing

For the time domain analysis of the MTR’s vibration, the crosstalk is removed in post-processing. This is achieved with the synchronous acquisition of the forcing signal and the output signal of the membrane: these are later subtracted taking into account the crosstalk, which is measured through a lock-in amplifier with the device under atmospheric pressure.

### Image processing

The image processing is performed using the difference of the resonance frequencies upon illumination and in dark condition. The resonances are identified with the maxima of the modulus of the signal derivative. The frequency-to-pixel position mapping is obtained through single pixel illumination with a laser.

## Supplementary information

Supplementary Information for: Room-temperature highly dispersive multiplexed device array for spatially resolved sensing and actuation
