# Supplementary material for: Highly dispersive multiplexed micromechanical device array for spatially resolved sensing and actuation
Source: Microsyst Nanoeng. 2024 Nov 26;10:179. doi: 10.1038/s41378-024-00816-z (PMC11599719; doi:10.1038/s41378-024-00816-z)
Supplement: Supplementary file 1 — Supplementary Information for: Room-temperature highly dispersive multiplexed device array for spatially resolved sensing and actuation [file 41378_2024_816_MOESM1_ESM.docx]

Supplementary Information for:

Highly dispersive multiplexed micromechanical device array for spatially resolved sensing and actuation

Leonardo Gregorat^1, ∗^, Marco Cautero^1, 2^, Leonardo Vicarelli^3^, Dario Giuressi^4^, Alvise Bagolini^5^, Alessandro Tredicucci^3^, Giuseppe Cautero^4^, and Alessandro Pitanti^3,6^

*^1^Department of Engineering and Architecture, Università degli Studi di Trieste, Trieste, Italy*

*^2^Department of Physics, Università degli Studi di Trieste, Trieste, Italy*

*^3^Department of Physics, Università di Pisa, Pisa, Italy*

*^4^Elettra Sincrotrone Trieste, Trieste, Italy*

*^5^Center for Sensors and Devices, Fondazione Bruno Kessler, Trento, Italy*

*^6^NEST Lab, CNR - Istituto di Nanoscienze and Scuola Normale Superiore, Pisa, Italy*

# S1. Fabrication

The Micromechanical Trampoline Resonators (MTRs) have been fabricated in a custom-made silicon nitride layer LPCVD-deposited on the two sides of a 500 μm thick Si wafer. The Si_3_N_4_ was deposited at different temperatures in order to achieve a medium tensile stress and improve the fabrication yield. In particular, our device layer consisted of two 105 nm thick low-stress Si_3_N_4_ layers (deposition T = 780 °C) which sandwiched a 90 nm thick high-stress Si_3_N_4_ one (deposition T = 760 °C). The final estimated tensile stress was around 560 MPa, as found by measuring the bending of test string devices. The fabrication started by defining a central circular region and side contacts via optical lithography (laser writer MicroWriter ML3), thermal metal evaporation and lift-off, resulting in a final layer of 3/50 nm of Cr/Au. A second aligned lithography was then used to define the membrane body, comprised of four tethers of variable width sustaining a central region of 100 μm lateral size. The whole membrane frame was chosen to be 300 μm. The optically lithographed pattern was transferred on the device layer via Cf_4_/H_2_-based reactive ion etching [1]. Finally, the devices were suspended via wet-etching in a 80° C KOH solution at 30% in water. S1818 was used as resist mask for the etching process, while LOR3A and S1805 bilayer were used for the lift-off process. Fig. S1 show colorized SEM images of a single device (a) and of the whole matrix array (b).


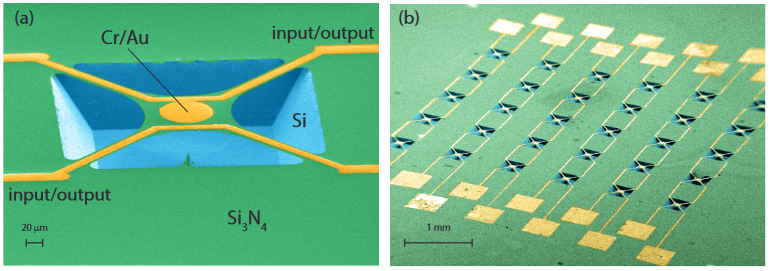


Fig. S1. False-color SEM image of a single device (a) and of the whole matrix array (b).

# S2. Experimental setup


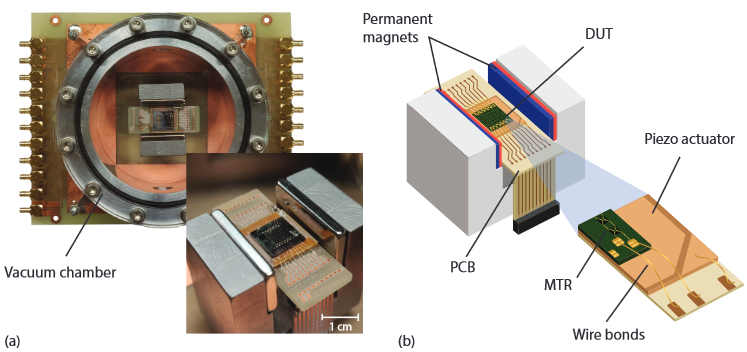


Fig. S2. (a): Picture of the custom vacuum chamber housing a matrix of MTRs. (b): 3D sketch of the chip mounted on the piezo actuator inside the magnetic field.

The custom vacuum chamber used is visible in Fig. S2 (a). The chamber was enclosed by an acrylic glass front panel and a custom printed circuit board (PCB) as the back panel. The PCB acted as a carrier board for a smaller PCB, which held the devices into the magnetic field. The piezoelectric actuator, by Physik Instrumente, was fixed to the daughter board with an indium alloy, while the chip containing the devices, which measured 1 cm^2^, was glued with an epoxy resin to the actuator. The devices were wire-bonded to the PCB, which passed on the signals to the carrier board, as in Fig. S2 (b). A U-shaped soft iron bracket embedding two Nd permanent magnets was used to produce the static and planar magnetic field. The produced magnetic field, measured by a calibrated gaussmeter, was ∼250 mT at the chip’s center and ∼300 mT at its edge.

The membrane vibration, as described in the main manuscript, can be induced by means of two techniques: by driving the piezoelectric actuator underneath the chip or by using the Lorentz’s force produced by an AC current passing through the membrane’s wire. The simplified electrical configurations used for the two techniques with a linear array are visible in Fig. S3 (a) and (b) respectively. The factor 126 high-impedance input voltage amplifier was used to amplify the sub-mV signal from the MTRs. The initial measurements on the MTRs were performed with commercial instruments: frequency sweep capable lock-in amplifier (Zurich Instruments UHFLI), oscilloscope (Teledyne LeCroy HDO9304) and arbitrary waveform generator (Siglent SDG6052X). To simplify the measurement setup while obtaining comparable or better result a custom FPGA-based all-in-one instrument tailored for this application was developed and used. All the results presented in the main manuscript were acquired with this custom electronics. The simplified block diagram of the instrument is visible in Fig. S3 (c). The use of a numerically controlled oscillator (NCO) allowed the generation of sample by sample deterministic, reproducible and configurable modulations. Moreover, the custom lock-in amplifier allowed access to the raw data coming from the ADC and the excitation signal, in order to obtain both time and frequency domain information.

The optical setup used for the single pixel illumination experiment is represented in Fig. S4 (a). The experiment was performed using a 445 nm laser pointer passing through a ∼700 μm pinhole, resulting in a ∼300 μW beam spot on the sample surface (measured with Thorlabs’ PM100D and S120VC). Using a 2D micrometric stage the laser spot was moved on the sample surface, allowing single pixel illumination. Through a raster scan this setup allowed to identify the mapping between the spatial position and the resonance frequency of each pixel. This was needed since, as stated in the main manuscript, there was a non-rigid frequency shift of the resonances, mostly due to device aging and not-uniform relaxation of the three silicon nitride layers.

The optical setup used for the matrix imaging experiment is visible in Fig. S4 (b). The source for this experiment was visible light produced by a RND Lab LED torch (700 lm) placed at 12 cm from the device’s surface. The device was masked with diagonally placed black tape, obstructing the light.


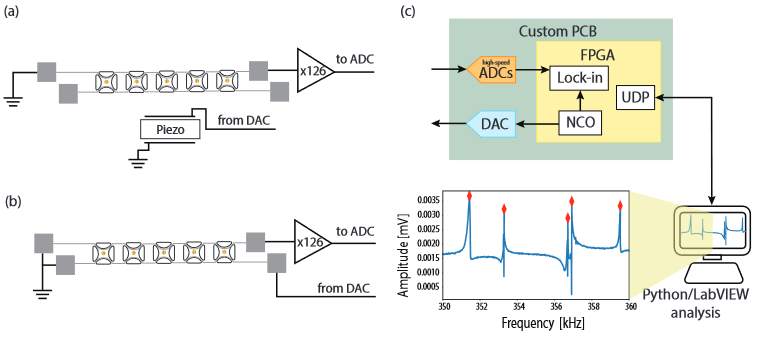


Fig.S3. Electical configuration for the readout of a linear array of MTRs excited by the piezoelectric actuator (a) and by the Lorentz induced force (b). (c): Simplified block diagram of the custom readout electronics.


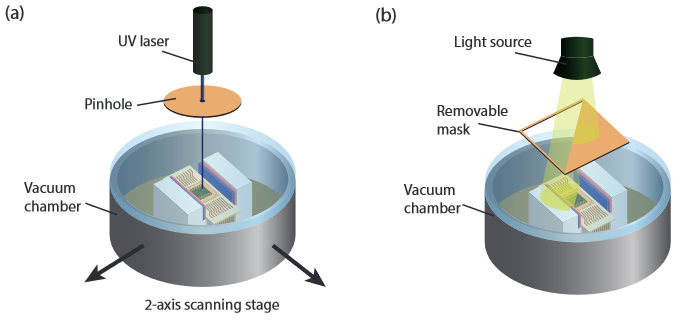


Fig. S4. 3D sketch of the setup for the single pixel characterization (a) and for imaging acquisitions (b).

# S3. Modeling and simulations

- Finite-element method simulations

The MTR eigenfrequencies were designed using a commercial finite-element method solver (Comsol Multiphyics). The multilayer device material was simulated as a single, 300 nm thick Si_3_N_4_ layer with 560 MPa initial stress. The metallic contacts were described by a 50 nm thick gold layer. The material parameters used in the simulations are reported in the following table:

| Material | Young’s modulus | Poisson’s ratio | Density |
| --- | --- | --- | --- |
| Si_3_N_4_ | 250 MPa | 0.23 | 3100 kg/m3 |
| Au | 70 MPa | 0.44 | 19300 kg/m3 |

The hanging frame of the trampoline was 300 μm and the length of the central plate was 100 μm. Every angle was opportunely filleted to avoid points with high intrinsic stress. The central disc absorber was a circle of 55 μm diameter and the metallic wires on the membrane were 10 μm wide. Finally, each MTR had a different tether width, linearly interpolated from 10 to 20 μm.

- Lorentz’s force differential equations modeling

The main manuscript reports the differential equations system used to reproduce the main features of the magnetomotive readout with piezoelectric forcing. In the case of Lorentz’s force actuation, we solved a single differential equation for the membrane displacement x_m_(t):

$\frac{\partial\dot{x}_{m}}{\partial t}= -\frac{\gamma_{m}}{2}\dot{x}_{m}(t)-\omega_{m}^{2}x_{m}\left( t \right)-d_{m}x_{m}\left( t \right)^{3}+K_{L}A_{F}sin(\omega_{F}t)$ (1)

where γ_m_ is the membrane dissipation, ω_m_ its eigenfrequency, dm the Duffing coefficient. The forcing at a frequency ω_F_ had an amplitude A_F_. K_L_ is the scaling factor for the input signal and takes into account contributions such as the length of the wire, its resistance and the magnetic field. The readout signal is then given by the magnetomotive term plus a contribution coming from the wire-to-wire crosstalk, i.e. η_ct_A_F_ sin (ω_F_ t + ϕ_ct_), η_ct_ and ϕ_ct_ being respectively the coupling efficiency and phase. Numerically solving Eq. (1) for different driving frequencies and demodulating the corresponding magnetomotive signal and the crosstalk produces the results of Fig. 2 (f) in the main manuscript.

- Magnetomotive readout

An estimate for the order of magnitude of the magnetomotive voltage can be done considering the fundamental mode of vibration of a simple one-dimensional string of length L. For small oscillations, its displacement is given by [2]:

$y(x, t) = C \cdot sin( \frac{\pi}{L} x) cos(\omega t)$ (2)

with C being the peak motional displacement. It is easy to see that the magnetic flux for a homogeneous field B orthogonal to the string displacement is given by:

$\Phi= B\int_{0}^{L} y(x, t) dx = BC \cdot cos(\omega t) 2L\pi$ (3)

and therefore, the magnetomotive voltage:

$V_{mm} = \dot{\Phi} = \omega BC \cdot sin(\omega t) 2\frac{L}{\pi}$ (4)

which, in our demodulated experiment, simply reads as V_mm_ = ωBC · 2L/π. Given the voltage measured in the experiment, we found the constant C which represents the motional displacement of the mechanical resonator.

# S4. Device nonlinearities

As mentioned in the main manuscript, the principal device nonlinearity comes from the Duffing term, which is commonly seen in micromechanical resonators, and originating further expanding the generalized Hooke’s law at a further order in the spring constant. The Duffing term causes a nonlinear softening/hardening that scales with the third power of the displacement [2] and therefore becomes relevant at high driving range. Duffing effects can lead to bistability in the system while, at small displacements, cause an asymmetry in the resonant peak shape. Fig. S5 highlights the nonlinear effects in our MTRs for both driving methods. Spectra for a selected resonator at different driving amplitudes are reported in panels (a) and (b) for piezoelectric actuation and Lorentz’s forcing respectively. Fixing the input frequency, for example considering the black dashed line, the MTR output voltage scales nonlinearly, as shown in panels (c) and (d) respectively. The yellow line is the best fit of the data, using a second order polynomial function, f (x) = ax^2^ + bx + c. The best fit for the nonlinear contribution a has been computed and is equal to 0.185 and 0.0173 mV _rms_ ^-1^ respectively.


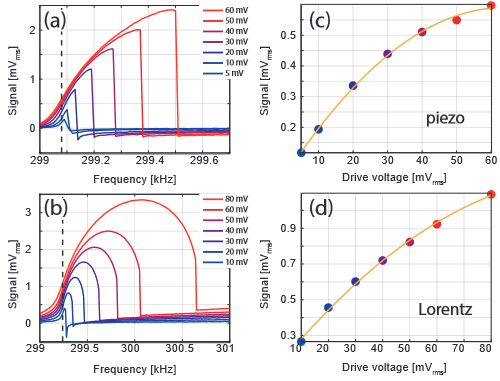


Fig. S5. Spectra of a selected MTR for increasing piezoelectric layer (a) and Lorentz’s force (b) drive amplitudes. (c-d): Signal at a specific frequency (dashed lines in the spectra) as a function of drive amplitude.

# References

[1] L. Vicarelli, A. Tredicucci, and A. Pitanti, Micromechanical bolometers for subterahertz detection at room temperature, ACS Phot. **9**, 360 (2022).

[2] S. Schmid, L. G. Villanueva, and M. L. Roukes, *Fundamentals of nanomechanical resonators*, Vol. 49 (Springer, 2016).
